# Effects of manipulated levels of predation threat on parental provisioning and nestling begging

**DOI:** 10.1093/beheco/arz060

**Published:** 2019-04-24

**Authors:** Ariane Mutzel, Anne-Lise Olsen, Kimberley J Mathot, Yimen G Araya-Ajoy, Marion Nicolaus, Jan J Wijmenga, Jonathan Wright, Bart Kempenaers, Niels J Dingemanse

**Affiliations:** 1 Research Group Evolutionary Ecology of Variation, Max Planck Institute for Ornithology, Seewiesen, Germany; 2 Department of Behavioural Ecology and Evolutionary Genetics, Max Planck Institute for Ornithology, Seewiesen, Germany; 3 Department of Biology, Center for Biodiversity Dynamics, Norwegian University of Science and Technology (NTNU), Trondheim, Norway; 4 Department of Biology, Behavioural Ecology, Ludwig Maximilian University of Munich (LMU), Planegg-Martinsried, Germany

**Keywords:** life-history trade-off, offspring provisioning, parental care, parental investment, predation risk, risk-taking

## Abstract

Parental provisioning behavior is a major determinant of offspring growth and survival, but high provisioning rates might come at the cost of increased predation threat. Parents should thus adjust provisioning activity according to current predation threat levels. Moreover, life-history theory predicts that response to predation threat should be correlated with investment in current reproduction. We experimentally manipulated perceived predation threat in free-living great tits (*Parus major*) by presenting parents with a nest predator model while monitoring different aspects of provisioning behavior and nestling begging. Experiments were conducted in 2 years differing greatly in ecological conditions, including food availability. We further quantified male territorial aggressiveness and male and female exploratory tendency. Parents adjusted provisioning according to current levels of threat in an apparently adaptive way. They delayed nest visits during periods of elevated perceived predation threat and subsequently compensated for lost feeding opportunities by increasing provisioning once the immediate threat had diminished. Nestling begging increased after elevated levels of predation threat, but returned to baseline levels by the end of the experiment, suggesting that parents had fully compensated for lost feeding opportunities. There was no evidence for a link between male exploration behavior or aggressiveness and provisioning behavior. In contrast, fast-exploring females provisioned at higher rates, but only in the year with poor environmental conditions, which might indicate a greater willingness to invest in current reproduction in general. Future work should assess whether these personality-related differences in delivery rates under harsher conditions came at a cost of reduced residual reproductive value.

## Teaser

Parental provisioning increases offspring survival but might attract predators towards adults and offspring. We show that parent birds reduce provisioning initially in response to a model nest predator, but compensate for lost feeding opportunities once the immediate threat has diminished. Mothers that worked harder to provision young at higher levels were also the ones more willing to take risks in nonprovisioning contexts, suggesting individual differences in how the trade-off between current and future reproduction is resolved.

## INTRODUCTION

In altricial bird species, parental provisioning is crucial for offspring growth and survival (Naef-Daenzer and Keller 1999; Metcalfe and Monaghan 2001), but it might also come at the cost of an increased threat of offspring and adult predation (Skutch 1949). This is because higher nest visitation rates and higher parental activity might increase the likelihood of attracting predators of either nestlings or parents (Skutch 1949; Martin et al. 2000b). To maximize reproductive success, parents should trade-off the probability of nestling starvation against the potential costs of nest predation and adult injury or death (Skutch 1949; Martin et al. 2000a; Martin et al. 2000b; Ghalambor and Martin 2001). It has previously been shown that parent birds are indeed capable of assessing potential threats and adjusting their behavior accordingly (Mahr et al. 2015). Parents can thus resolve the trade-off by adjusting provisioning activity according to the level of nest predation threat experienced during provisioning (Lima 2009; Martin and Briskie 2009), while also taking into account hunger level and general condition of their nestlings to balance the costs of the provisioning interruptions. For instance, parents reduce offspring provisioning under high levels of predation threat, and then compensate for lost feeding opportunities by subsequently increasing nest visit rates and/or delivering larger loads per visit once the immediate threat has diminished (Tilgar et al. 2011; Mutzel et al. 2013a; Moks et al. 2016).

Most studies to date have focused on immediate parental responses towards an increased predation threat (e.g., Ghalambor and Martin 2001; Eggers et al. 2005; Peluc et al. 2008), but little is known about how parents adjust different aspects of their provisioning behavior following an increase in predation threat. This is surprising given that prolonged provisioning interruptions during periods of high levels of perceived predation threat should result in an increased nestling demand (Leonard and Horn 1996) and louder begging of hungry nestlings, attracting the attention of predators (McDonald et al. 2009b). The way parents cope with such increased demand is thus likely to affect nestling growth and survival and consequently parental reproductive success. Two previous studies, one on blue tits (*Cyanistes caeruleus*) and one on pied flycatchers (*Ficedula hypoleuca*), showed that parents decreased their nest visitation rates under high levels of perceived predation threat and subsequently increased feeding rates above baseline levels once the immediate threat had diminished (Mutzel et al. 2013a; Moks et al. 2016). However, neither study differentiated between parental responses caused by human disturbance at the nest per se and the presentation of a harmless object or a predator model. Furthermore, Moks et al. (2016) did not control for baseline feeding rates. An improved experimental design is therefore required to provide a full picture concerning the actual cues parents respond to and to reveal the strength of responses towards different levels of disturbances. Moreover, monitoring visit rates while neglecting other aspects of provisioning behavior might not give a full understanding of parental adjustments and responses towards different levels of perceived predation threat. This is because it is likely that parental responses involve not only changes in feeding rate, but also changes in the size or the type of prey delivered to the nest (Wright et al. 1998; Mathot et al. 2017). This implies that nest visitation rates might not always be a good predictor of the actual amount of food, nutrients, and energy delivered to the nest (e.g., Bengtsson and Rydén 1983; Blondel et al. 1991; Wright et al. 1998; Naef-Daenzer and Keller 1999; Grieco 2001).

To better understand how changes in parental provisioning affect nestling condition, as well as the mechanisms mediating any subsequent changes in parental behavior, it is useful to quantify multiple aspects of parental responses along with any temporal changes in nestling need. Nestling begging intensity is influenced by nestling hunger and condition (Kilner 1995; Cotton et al. 1996; Kölliker et al. 1998), since it increases with food deprivation (Leonard & Horn 1996) and can thus be considered as an honest signal of nestling long- and short-term need (Price et al. 1996; Wright et al. 2010a). Moreover, several studies have shown that nestling begging intensity directly affects parental effort by increasing parental provisioning rates and/or increasing load sizes (Wright and Cuthill 1990; Ottosson et al. 1997; Wright 1998; Kilner et al. 1999; Hinde 2006; Wright et al. 2010b). Begging intensity might thus represent a potential mechanism by which increases in parental provisioning can be mediated after periods of feeding interruptions due to a change in perceived predation threat (Mutzel et al. 2013a). Simultaneous monitoring of multiple aspects of provisioning behavior and nestling begging will give detailed insights into 1) the behavioral mechanisms parents adopt to manage conflicting demands during varying levels of perceived predation threat and 2) whether such adjustments can help mitigate potential harmful short- and long-term consequences for nestling growth and survival.

Although the study of population-level responses can demonstrate how the average individual responds to changes in perceived predation threat, only individual-level studies can reveal differences in how individuals resolve important life-history trade-offs, for example, between their own and nestling survival and/or current and future reproduction (Duckworth 2006; Wolf et al. 2007; Smith and Blumstein 2008). For instance, some individuals might more readily accept the costs associated with certain behaviors and therefore be better at acquiring and defending resources (Cole and Quinn 2012). This in turn might enable them to increase their investment in the current brood, for example, by provisioning nestlings at higher rates. Nevertheless, engaging in costly behaviors is likely to come at the expense of a decreased lifespan and therefore reduced residual reproductive value (Smith and Blumstein 2008; Réale et al. 2010). Individual differences in how the trade-off between current and future reproduction is resolved are thus expected to covary with individual differences in various behavioral traits related to risk-taking, such as exploration behavior and aggressiveness (Stamps 2007; Wolf et al. 2007; Dammhahn et al. 2018; Jablonszky et al. 2018; Mathot and Frankenhuis 2018; Wright et al. 2019).

Here, we investigate how parent great tits (*Parus major*) adjusted aspects of parental care (including feeding latency, inter-visit interval, load size, delivery rate, and prey type) in response to experimentally manipulated levels of perceived predation threat. We did this by presenting pairs of great tits with a taxidermic model of a great spotted woodpecker (*Dendrocopos major*) while simultaneously monitoring parental provisioning behavior and nestling begging intensity before, during and after the presentation. The great spotted woodpecker is a common nest predator of Eurasian cavity nesting birds (Löhrl 1972) and is a common source of nestling mortality and nest failure in great tit populations. In our great tit population, great spotted woodpeckers are frequently observed at nestboxes, trying to access the nest and even successfully removing nestlings (Mutzel A, personal observations). Even though woodpeckers are usually not a threat to adult birds (Curio 1975), they might still harm adult parents caught inside the nest cavity (Mutzel A, personal observations). Pilot experiments showed that parents responded to the presence of the woodpecker model with enhanced nest defense behavior and an interruption of nestling feeding, implying that our experiment successfully increased the perceived predation threat. To investigate whether parents recognized the woodpecker model as a potential predator or were merely responding to the sudden appearance of another species in front of their nestbox, we also presented a taxidermic model of a harmless nonpredator species, the common blackbird (*Turdus merula*). We further explored how much of the observed parental responses were due to the short human disturbance at the nestbox, when placing or removing the models, because human presence itself might also be perceived as a predation threat for both parents and offspring. We then explored whether individual provisioning responses to experimental changes in perceived predation threat were associated with individual variation in 2 behavioral traits commonly used as proxies for risk-taking, male territorial aggressiveness, and male and female exploration (e.g., Hollander et al. 2008; Quinn et al. 2012; Stuber et al. 2013; Cole and Quinn 2014).

The study was carried out in 2 years that differed substantially in ecological conditions, including temperature, precipitation, and food availability (Nicolaus et al. 2015; Mathot et al. 2017), which resulted in significant between-year differences in nestling condition and nestling mortality (Nicolaus et al. 2015). This suggests that the costs and benefits of provisioning behavior for parents differed between years, with higher costs in the year with low-food availability when parents presumably had to work harder to find food. We therefore predicted that parental responses towards different levels of perceived predation threat might differ between years. We predicted specifically that 1) parents interrupt provisioning for some time during increased levels of perceived predation threat to prevent nest predation, with a weaker effect during the year with more challenging ecological conditions. This is because feeding interruptions should have more detrimental effects on nestlings that are in poorer condition. We also predicted that 2) parents increase their food delivery rates above baseline levels once the immediate predation threat has diminished, to compensate for lost feeding opportunities earlier on. This effect should be less pronounced in the “bad” year, because parents’ baseline provisioning should have been already closer to their maximum sustainable rate. Finally, we predicted that 3) aggressive and explorative individuals deliver food at higher rates under baseline conditions and after increased levels of predation threat. This is because a higher provisioning effort is likely to increase the probability of mortality (Clutton-Brock 1991; Royle et al. 2012), for example, through increased threat of predation or physiological aging (Nilsson 2002; Tilgar et al. 2011) and can thus be considered as a costly type of behavior. We expected this correlation to be stronger in the “bad” year when high levels of provisioning could presumably only be achieved at the expense of self-foraging or self-maintenance and/or by accepting a higher chance of mortality while foraging.

## MATERIAL AND METHODS

### Study site and general field procedures

The study was carried out in 12 nestbox plots of great tits located south of Munich in Southern Germany (47°55′–48°01′N, 11°09′–11°20′E), during the breeding seasons of 2010 and 2011. Each plot consisted of 50 nestboxes resulting in a total of 600 nestboxes. From early April till the end of the breeding season (August), we checked nestboxes at least twice per week to record lay date, onset of incubation, hatching and fledging date, and the number of young fledged. As part of another study, we manipulated brood sizes such that individuals had to raise either a reduced (net decrease of 3 nestlings), a control (no net change in brood size but swapping of about half of nestlings), or an enlarged brood (net increase of 3 nestling), when nestlings were 3 days old (for more details see Nicolaus et al. 2015). When nestlings were 7 or 11 days old, we caught both parents inside the nestbox. Individuals that were not previously banded were fitted with a numbered metal band and a unique combination of 3 color bands.

### Perceived predation threat experiment

Provisioning behavior of both parents was recorded at 88 nestboxes (*N*_2010_ = 41; *N*_2011_ = 47) with reduced (*N* = 26; mean brood size: 4.5, range: 2–7), enlarged (*N* = 28; mean: 8.4, range: 4–13), or control broods (*N* = 34; mean: 4.5, range: 5–11; see above). The experiment was carried out between 0800 and 1300 h when nestlings were 12 days old, the age at which parental provisioning rates are highest (Gibb 1955; Verhulst and Tinbergen 1997; Barba et al. 2009). Out of the 176 individuals, 12 were recorded in both years, resulting in 164 unique individuals over both years. Two days before the experiment, we installed an infrared camera (CDD Bird Box Camera with IR Night Vision 420TV lines) by exchanging the side door of the nestbox with a small wooden box containing the camera, to allow parents to habituate to the setup. The nestbox and the camera-containing box were separated by a plexiglass partition. We raised each nest cup by inserting a 2-cm thick piece of foam underneath the nest material, to ensure that the camera captured the entire nest cup. The following day we installed a wooden, 1.5-m high pole 2 m from the entrance of the target nestbox, on which the models would be fixed during the experimental treatments, to habituate parents to the presence of this object. We did not observe cases of nest abandonment after raising the nest cup and installing a nestbox camera.

**Figure 1:**
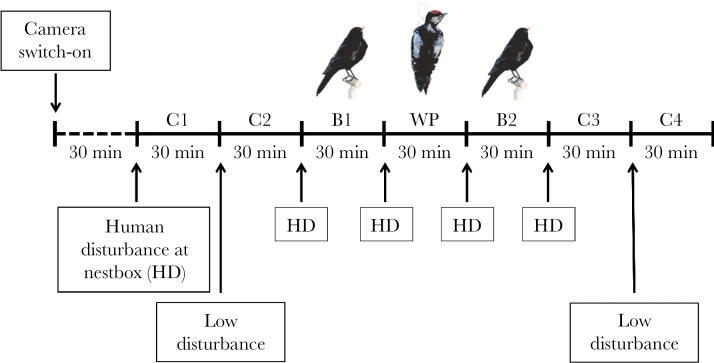
Setup of the perceived predation threat experiment (C = control, B = blackbird, and WP = woodpecker presentation at the nestbox). HD refers to high levels of disturbance at the nestbox at the beginning of those treatments.

On the day of recording (nestling age 12), we connected the nestbox camera to a portable recording device (Archos 5 Internet Media Tablet) and a power supply placed at a distance of 20 m from the nestbox. After switching on the camera, we left the immediate nest environment (>100 m) to allow parents to recover from the disturbance. Half an hour later, we started the actual experiment, which comprised 7 consecutive trials approximately 30 min in length (Figure 1). The first 2 trials consisted of control treatments, which differed in whether the parents were subject to human disturbance at the nestbox (C1) or at a distance of 20 m (C2). The next trial served as a control for the appearance of a familiar but harmless bird, a common blackbird, close to the nestbox (B1). The fourth trial represented the actual increased predation threat treatment (WP), where we presented a model of a potential nest predator, the great spotted woodpecker. During the fifth trial, we presented a blackbird model again (B2) to control for potential sequence effects of the model presentations. The last 2 trials represented further control treatments: human disturbance at the box (C3) and at 20 m (C4). We used 9 different woodpecker and 9 blackbird models, which were randomly assigned to the different nests to avoid problems associated with pseudo-replication (Kroodsma et al. 2001). Neither the woodpecker nor blackbird model identities explained variation in any aspects of the parental responses described below (Supplementary Material).

During the low human disturbance trials (C2 and C4), the observer walked up to the recording device, located 20 m from the nest, stopped and immediately restarted the video recording, and left the immediate nest environment again (>100 m) for the entire duration of the trial. For the trials with human disturbance at the box (C1), the observer walked up to the nestbox and placed and/or removed a stuffed model (B1, WP, B2, C3), before restarting the recording and then leaving the immediate nest surroundings. This resulted in brief interruptions between consecutive 30-min trials that slightly differed across nests (average duration of disturbance: 83.6 s). There was a small effect on parental latencies to return to the nestbox for C2 and C4 (C2: β = 0.0018, 95% CI: 0.0000, 0.0003; C4: β = 0.0016, 95% CI: 0.0002, 0.0028), but not for the other trials. Furthermore, the duration of the interruptions had only a minor nontreatment-specific effect on subsequent provisioning behavior. It thus seems unlikely that treatment-specific differences in provisioning behavior were strongly affected by differences in the duration of the interruption. By comparing parental behavioral patterns between the different experimental and control treatments, this experimental setup allowed us to differentiate between effects caused by human presence at the nest itself (C1), distant human presence (C2), the additional presence of a nonthreatening bird (B1), and a potential nest predator (WP) and to investigate potential carry-over effects of the woodpecker presentation (B2, C3, and C4).

At the onset of each trial, we additionally recorded whether at least 1 parent was alarm calling. The presence of one of the parents at the beginning of a trial had a highly significant negative effect on feeding latency (β = −0.48, 95% credible interval [CI]: −0.55, −0.41). However, adding “presence” of parents (yes/no) as additional fixed effect to the models described below did not affect any of the conclusions (results not shown). Thus, to simplify the model structure, this variable was not included in the main models presented below.

This experimental setup resulted in a total of 616 trials (7 trials for each of the 88 nestboxes). However, the final sample size was slightly reduced as we could not collect behavioral data for 12 trials (i.e., <2%), because the video file was corrupted or because of technical failure of the recording device.

### Parental provisioning behavior

We analyzed provisioning behavior during the first 30 min of each treatment, although duration of trials differed slightly for logistical reasons (mean: 32 min; range: 30–46 min). For each parental visit, we retrieved the following variables from the videos: bird identity, nestbox entrance and exit times, load size (visually estimated relative to the adult’s bill volume (e.g., 1 = volume of bill, 2 = twice the volume of bill, scored to the nearest 0.1), and prey-type category defined as preferred (caterpillars and spiders) versus nonpreferred (all other prey types) (Naef-Daenzer et al. 2000; Wilkin et al. 2009). We used these data to calculate inter-visit intervals (IVI) and short-term delivery within each treatment for each individual. IVIs were defined as time elapsed between 2 successive visits of the same individual and were calculated as nestbox entry time of visit *N* minus nestbox exit time of visit *N* − 1. Individual short-term delivery was estimated as load size/IVI, which integrates the amount of food delivered to the brood per visit with the elapsed time since the last (individual) feeding event and thus reflects the amount of food a brood received per unit time.

In addition to these visit-specific variables, for which multiple measures were available for each individual and treatment, we also calculated variables with only one measure per treatment and individual: “latency to restart feeding,” defined as time elapsed between the onset of each trial and an individual’s first nestbox visit, and “long-term delivery” calculated as the sum of all load sizes delivered to the nest during a trial over the total observation time. Long-term delivery integrates provisioning interruptions at the beginning of a trial, the subsequent feeding rate, and the amount of food delivered during each visit and is therefore the best estimate of how much food the nestlings received during an entire trial. Individuals that did not return to the nestbox for an entire 30-min trial were assigned a feeding latency of 1801 s and a long-term delivery of 0 for this trial (*N* = 110 out of 1208). Rerunning models without these individuals did not affect the results (details not shown).

### Nestling begging

We scored begging levels of individual nestlings for every parental feeding visit at the exact time each parent entered the nestbox. We did this by recording how high each nestling begged relative to its own height at rest, on a scale from 0 to 10 (e.g., 0 = no begging/resting, 10 = full body height of a 12-day-old nestling with fully extended body and legs—see Wright et al. 2002). Previous studies have shown that begging posture or height is a reliable indicator of nestling nutritional need (Kilner and Johnstone 1997; Wright and Leonard 2002; Wright et al. 2010a) and they are tightly correlated with acoustic measures of begging intensity in our great tit population (both within and among broods, Olsen A-L, unpublished data) and in other cavity-nesting species (Kilner 2002; Leonard et al. 2003; Wright et al. 2010b). The correct scoring of begging levels required extensive training of observers (*N* = 7 observers in our case). Observers were trained until within- and between-observer correlations were greater than 0.80 (average between-observer correlation: 0.91, range: 0.84–0.97; average within-observer correlation: 0.96, range: 0.91–0.99). We then calculated average brood begging intensity (sum of begging level of all nestlings/brood size) for every parental visit.

### Male aggressiveness

Male territorial aggressiveness was measured 4 times per individual per brood, twice during egg laying and twice during incubation, using a simulated territorial intrusion test. For this study, we used data from 78 unique individuals tested for 82 experimental broods (2010: 39 individuals; 2011: 43 individuals; 4 individuals were tested in both years; not including 6 males that did not respond during any of the 4 aggression tests). A taxidermic mount of a male great tit with great tit song playback was presented in front of the focal male’s nestbox. Once the male entered a 15-m radius around the nestbox, its behavior was recorded for 3 min. If a male did not respond within the first 15 min, we scored the male as nonresponding. We used the average minimum approach distance over all responsive tests as a proxy for male aggressiveness (for a more detailed description of the setup, see Araya-Ajoy and Dingemanse 2014). To control for effects of nest stage on aggressiveness, we mean-centered the minimum approach distance within nest stage and year before calculating the average score per individual and brood. The final score was multiplied by −1 such that higher scores reflected higher levels of aggression.

### Exploration behavior

We measured exploration behavior of parents captured inside their nestbox (at nestling age 7 or 11 days). Each individual was tested once a year. We used exploration behavior data from 168 observations (*N*_2010_ = 77; *N*_2011_ = 91) of exploration behavior for 157 unique individuals of the 88 experimental broods (11 individuals were tested in both years). Directly after capture, birds were transferred to a small compartment connected to the “exploration cage” (61L × 39W × 40H cm) via a sliding door. After 1 min of acclimation, the bird was released into the cage without handling and its behavior was filmed for 2 min. For the video analysis, we divided the experimental cage into 6 zones (see Stuber et al. 2013 for graphical illustration). Exploration behavior was then scored as the total number of movements across different zones, excluding minor position changes within zones and reflects a personality-related measure. For later analysis, we divided this score by 120 to obtain the number of movements per second.

### Statistical analysis

#### Effects of treatment on parental behavior and nestling begging

To investigate how treatment affected initial responses (feeding latency), subsequent provisioning behavior (IVI, load size, short-term delivery, and prey type), long-term delivery (an integrated measure for latency and short-term delivery) of each parent and nestling begging, we analyzed sources of variation in these variables using linear mixed-effects models with treatment (factor with 7 levels: C1, C2, B1, WP, B2, C3, or C4), year (factor with 2 levels: 2010 or 2011), brood size (at the time of the experiment; within-year mean-centered), and parental sex as fixed effects. To explore year-specific effects of treatment, initial models also included the interaction between treatment and year. We further fitted nest identity, individual identity (except for models of nestling begging), and trial identity (defined as the unique combination of nest identity and treatment) as random effects. Individual identity (*N* = 164) was nested within nest identity (*N* = 88) to acknowledge that the behavior of males and females could be influenced by factors associated specifically with the nestbox location. Trial identity (*N* = 604) was nested within individual and nest identity to allow estimating the effects of treatment without pseudo-replication (i.e., controls for repeated observations within each 30-min trial over and above the effects of nest or individual identity). It also controlled for trial-specific effects, caused by among-trial variation in factors such as weather or the behavior of the partner. Rather than investigating the separate effects of natural brood size and brood size manipulation (as in Nicolaus et al. 2015), we simply controlled for current brood size. This simplification seems justified since separate analyses carried out using natural brood size and brood size manipulation yielded qualitatively similar results (Supplementary Tables S1 and S2).

Latency, IVI, load size, and short- and long-term delivery were log-transformed in all models resulting in residuals that approximated a Gaussian distribution. Prey type was modeled as a binomial variable (1 = preferred prey; 0 = non-preferred prey). For all initial models, we used the C2 treatment (low disturbance) as reference category as this allowed us to investigate effects of the different levels of disturbance during the other treatments on parental responses while controlling for baseline behavior. Twelve individuals were tested in both years, which allowed us to calculate the adjusted repeatability of feeding latency following the woodpecker presentation between years using linear mixed-effects models with random intercepts for individual and nest identity and brood size fitted as fixed effect.

#### Aggressiveness, exploration behavior, and provisioning responses

We investigated the link between male aggressiveness and male and female exploration behavior and parental short-term delivery. To evaluate whether male aggressiveness explained variation in parental delivery, we ran linear mixed-effects models with log-transformed short-term delivery as the response variable and with mean-centered aggressiveness, treatment, brood size (within-year mean-centered), and the interaction between aggressiveness and treatment as fixed effects. We further fitted random intercepts for individual and trial identity (nested within individual). We explored whether the effect of aggressiveness on delivery differed between years by fitting a 3-way interaction between aggressiveness, treatment, and year. Next, we investigated whether exploration behavior (mean-centered) explained variation in parental delivery using a similar model structure as described for aggressiveness, but with parental sex fitted as additional fixed effect and nest identity as additional random effect (as exploratory behavior was measured for both members of a pair). To explore year and sex differences, we also fitted a 4-way interaction between exploration, treatment, year, and sex.

All statistical analyses were performed in R version 3.4.2 (R Development Core Team 2017). Linear mixed-effects models were fitted with the “lmer” function (Bates et al. 2015). We simulated the posterior distributions of each fixed effect parameter using the “sim” function of the package arm (Gelman and Yu-Sung 2018) to estimate effect sizes and their 95% CIs. Estimates with CIs not including zero are considered as “strongly supported” or the presence of “strong evidence.” As this method provides separate estimates for each category of a factorial predictor variable making it difficult to judge its overall significance, we additionally used the chi-square distributed Wald statistic for calculating the overall significance of factorial variables (Fox and Weisberg 2011). Repeatability was calculated as the among-individual variance divided by the total phenotypic variance.

## RESULTS

### Year differences in provisioning behavior and nestling begging

Contrary to our prediction, feeding latencies due to disturbance at the nest in 2010 were not generally shorter compared with those in 2011 (Table 1). However, in 2011 parents delivered larger food items, had higher short- and long-term delivery rates (Tables 1 and 2), and had a higher probability of delivering preferred prey items (i.e., caterpillars and spiders; β = 2.41, 95% CI: 2.08, 2.77), consistent with results from previous studies which indicated that the foraging conditions were better in this year. There was also an overall effect of year on nestling begging, with nestlings begging more intensively in 2010 compared with 2011 (Table 1).

**Table 1:** Effect of treatment on 3 aspects of parental provisioning behavior and on nestling begging

	Log (Latency)	Log (Load size)	Log (Short-term delivery)	Begging
	β (95% CI)	β (95% CI)	β (95% CI)	β (95% CI)
Intercept	2.56 (2.48, 2.64)	0.27 (0.26, 0.29)	1.46 (1.42, 1.51)	4.81 (4.46, 5.16)
Treatment^a^				
C1	**0.11** (0.03, 0.19)	0.00 (−0.01, 0.02)	−0.01 (−0.05, 0.03)	0.04 (−0.17, 0.31)
B1	**0.16** (0.08, 0.24)	−0.01 (−0.02, 0.00)	−0.01 (−0.05, 0.02)	0.12 (−0.10, 0.38)
WP	**0.33** (0.24, 0.40)	0.00 (−0.02, 0.01)	−0.03 (−0.07, 0.02)	**0.41** (0.14, 0.67)
B2	**0.12** (0.05, 0.20)	0.00 (−0.01, 0.01)	0.03 (−0.01, 0.07)	**0.52** (0.24, 0.71)
C3	−**0.11** (−0.19, −0.03)	−0.01 (−0.03, 0.00)	0.02 (−0.01, 0.06)	0.09 (−0.14, 0.33)
C4	−**0.22** (−0.28, −0.13)	−0.01 (−0.02. 0.00)	**0.04** (0.01, 0.07)	−0.06 (−0.28, 0.20)
Sex male^b^	−0.03 (−0.08, 0.03)	0.01 (0.00, 0.02)	0.00 (−0.03, 0.04)	**0.31** (0.16, 0.42)
Year 2011^c^	−0.03 (−0.10, 0.05)	**0.09** (0.08, 0.11)	**0.10** (0.06, 0.14)	**−0.87** (−1.31, −0.43)
Brood size	**−0.05** (−0.07, −0.04)	0.00 (0.00, 0.00)	**0.03** (0.02, 0.04)	**0.17** (0.08, 0.28)
N	1208	8215	7119	7942

^a^Reference category is treatment “C2.”

^b^Reference category is sex “female.”

^c^Reference category is year “2010.”

Estimates were derived from linear mixed-effects models with random intercepts for nest (*N* = 88), individual (*N* = 164), and trial identity (*N* = 604). Treatment (7 levels), year (2 levels), sex, and brood size were fitted as fixed effects. C = control (C1 and C3: human disturbance at nestbox; C2 and C4: human disturbance at a distance of 20 m from nest), B = blackbird, and WP = woodpecker presentations in the order presented at the nestbox). Shown are point estimates for each fixed effect (β) with their 95% credible intervals (CIs). Effects that were strongly supported by the model have 95% CIs that do not overlap zero and are highlighted in bold.

**Table 2:** Effect of treatment on IVI and long-term delivery for 2010 and 2011

	Log (IVI)	Log (long-term delivery)
	β (95%CI)	β (95%CI)
2010		
Intercept	2.01 (1.95, 2.07)	0.77 (0.69, 0.88)
Treatment^a^		
C1	0.01 (−0.05, 0.08)	−0.09 (−0.18, 0.01)
B1	−0.02 (−0.08, 0.05)	**−0.12** (−0.22, −0.03)
WP	0.02 (−0.06, 0.08)	**−0.26** (−0.34, −0.15)
B2	−0.06 (−0.11, 0.01)	0.03 (−0.08, 0.11)
C3	−0.01 (−0.07, 0.05)	0.07 (−0.03, 0.17)
C4	−0.03 (−0.09, 0.04)	0.07 (−0.02, 0.18)
Sex male^b^	0.04 (−0.03, 0.09)	0.02 (−0.07, 0.10)
Brood size	−0.01 (−0.03, 0.01)	**0.06** (0.03, 0.09)
*N*	3351	557
2011		
Intercept	2.11 (2.05, 2.17)	0.90 (0.83, 0.98)
Treatment^a^		
C1	0.00 (−0.05, 0.07)	−0.03 (−0.12, 0.03)
B1	−0.01 (−0.08, 0.05)	−0.08 (−0.15, 0.00)
WP	0.03 (−0.05, 0.10)	**−0.31** (−0.39, −0.23)
B2	−0.03 (−0.10, 0.03)	−0.06 (−0.15, 0.01)
C3	**−0.10** (−0.17, −0.05)	**0.13** (0.04, 0.20)
C4	**−0.10** (−0.15, −0.03)	**0.14** (0.05, 0.20)
Sex male^b^	−0.01 (−0.05, 0.02)	**0.06** (0.01, 0.11)
Brood size	**−0.04** (−0.05, −0.02)	**0.07** (0.05, 0.09)
*N*	3788	648

^a^Reference category is treatment “C2.”

^b^Reference category is sex “female.”

Estimates were derived from linear mixed-effects models with random intercepts for nest (*N* = 88), individual (*N* = 164), and trial identity (*N* = 604). Treatment (7 levels), sex, and brood size were fitted as fixed effects. C = control (C1 and C3: human disturbance at nestbox; C2 and C4: human disturbance at a distance of 20 m from nest), B = blackbird, and WP = woodpecker presentations in the order presented at the nestbox). Shown are point estimates for each fixed effect (β) with their 95% credible intervals (CIs). Effects that were strongly supported by the model (95% CIs not overlapping zero) are highlighted in bold.

### Effects of treatment on feeding latency

**Figure 2:**
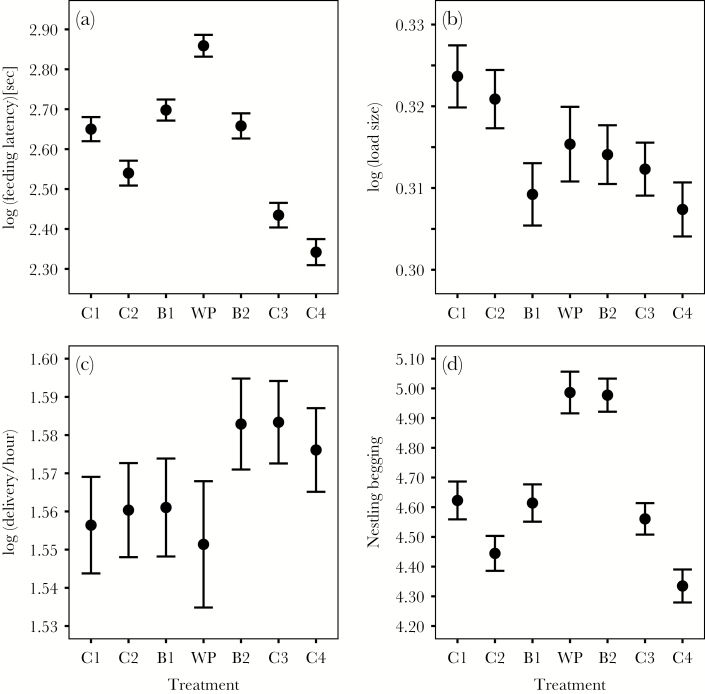
Effect of experimental treatment on (a) feeding latency, (b) load size, (c) short-term delivery, and (d) nestling begging. C = control, B = blackbird, and WP = woodpecker presentations at the nestbox in the order presented in the experiment. Shown are the means and standard errors of the raw values.

Analyses of feeding latencies revealed a strong effect of treatment (χ^2^_6_ = 233.64, *P* < 0.001), which did not differ between years (interaction treatment × year: χ^2^_6_ = 1.09, *P* = 0.98; Supplementary Figure S1a). As expected, feeding latencies after human disturbance at the nest (C1) were longer compared with human disturbance at a distance of 20 m (C2; Table 1, Figure 2). There was no additional effect of the blackbird presentation (B1 vs. C1: β = 0.03, 95% CI: −0.03, 0.12; Figure 2), indicating that great tits did not perceive the sudden appearance or the presence of a blackbird model close to their nest as an additional threat. In contrast, we found strong evidence that the presentation of the woodpecker (WP) prolonged parental feeding latencies over and above the effects of the brief human disturbance at the nest (WP vs. C1: β = 0.22, 95% CI: 0.13, 0.29; Figure 2) or the presence of a harmless bird species (WP vs. B1: β = 0.15, 95% CI: 0.08, 0.23; Figure 2). In addition, 23% of all individuals interrupted provisioning during the entire duration of WP, whereas this was only the case for 10% of all individuals during B1. To investigate potential carry-over effects of the woodpecker presentation, we compared feeding latencies during the treatments with similar levels of disturbance before and after WP. There was no evidence that feeding latencies during B2 differed from those during B1 (β = −0.04, 95% CI: −0.11, 0.04; Figure 2). In contrast, parents resumed feeding significantly sooner during the last 2 control treatments (C3 vs. C1: β = −0.22, 95% CI: −0.30, −0.14; C4 vs. C2: see Table 1; Figure 2). There was also a negative effect of current brood size on feeding latencies following disturbance (Table 1). Parents feeding larger broods resumed provisioning more quickly compared with parents with smaller broods. Feeding latencies during the woodpecker presentation were repeatable between years (*r* = 0.16; 95% CI: 0.14, 0.20).

### Effects of treatment on provisioning behavior

**Figure 3:**
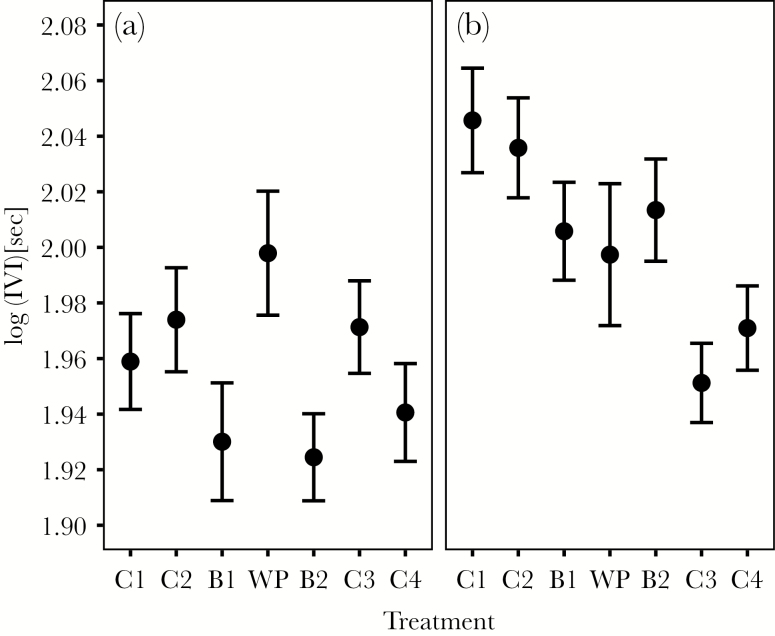
Effect of experimental treatment on IVI for (a) 2010 and (b) 2011. C = control, B = blackbird, and WP = woodpecker presentation at the nestbox in the order presented in the experiment. Shown are the means and standard errors of the raw values.

**Figure 4:**
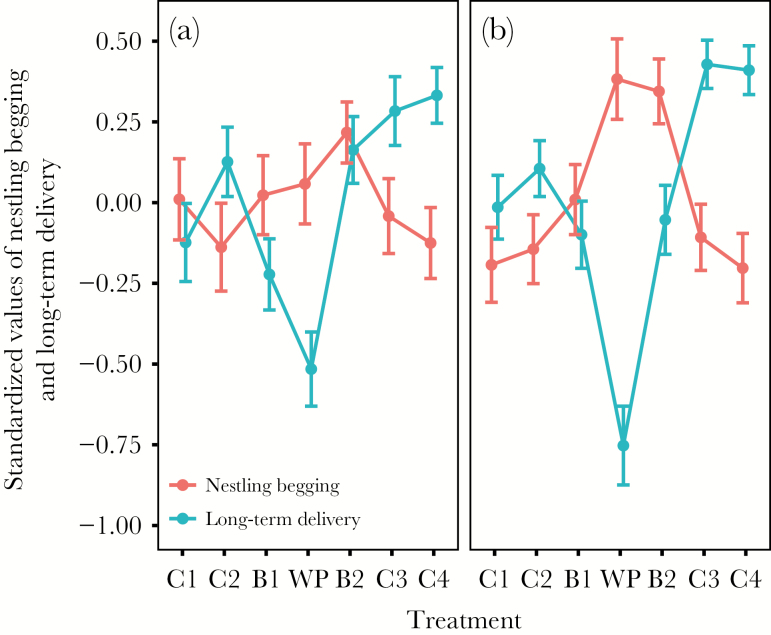
Effect of experimental treatment on long-term delivery and average nestling begging for (a) 2010 and (b) 2011. C = control, B = blackbird, and WP = woodpecker presentation at the nestbox in the order presented in the experiment. The graph depicts standardized values of both variables (to a mean of 0 and standard deviation of 1 within year) to allow direct comparison of the strength of the treatment effect across behavioral variables with different units. Dots show mean values and whiskers give standard errors.

We found strong evidence for an effect of treatment on IVI, load size, and short- and long-term delivery (IVI: χ^2^_6_ = 24.41, *P* < 0.001; load size: χ^2^_6_ = 13.70, *P* = 0.03; short-term delivery: χ^2^_6_ = 18.84, *P* = 0.004; long-term delivery: χ^2^_6_ = 256.77, *P* < 0.001; Figures 2–4). The effect of treatment on long-term delivery differed between years (year × treatment effect: χ^2^_6_ = 12.95, *P* = 0.04). There was also some support for a year-specific effect of treatment on IVI (year × treatment: χ^2^_6_ = 10.98, *P* = 0.08; Figure 3). There was no evidence for a year-specific treatment effect for load size (χ^2^_6_ = 8.18, *P* = 0.23) and short-term delivery (χ^2^_6_ = 8.53, *P* = 0.20; Supplementary Figure S1b,c). We did not find any evidence for a treatment-specific change in prey type (χ^2^_6_ = 8.78, *P* = 0.19; interaction treatment × year: χ^2^_6_ = 9.86, *P* = 0.13).

In both years, during the woodpecker presentation, parents fed broods at similar rates compared to the baseline rates during C2 once they had resumed provisioning (IVI: Table 2, Figure 3). In 2011, parents decreased their IVI (i.e., increased their feeding rates) and increased their long-term delivery during C3 and C4, whereas there was no such effect for 2010 (Table 2; Figures 3 and 4). This confirms our prediction that the increase in provisioning after high levels of perceived predation threat would be more pronounced in the “good” year. However, there was also a slight increase in short-term delivery rate during C4, which did not differ between years (Table 1; Figure 2).

### Effects of treatment on nestling begging

There was a strong effect of treatment on nestling begging (χ^2^_6_ = 29.51, *P* < 0.001) that did not differ between years (year × treatment: χ^2^_6_ = 3.21, *P* = 0.78). Nestlings begged at higher rates compared with baseline levels of C2 during and directly after the presentation of the woodpecker (WP and B2; Table 1; Figure 2) and B1 (B2 vs. B1: β = 0.35, 95% CI: 0.09, 0.57), but decreased their begging back to control levels during C3 and C4 (Table 1; Figure 2).

### Individual-specific responses related to aggressiveness and exploration behavior

Contrary to our prediction, we did not find any evidence for an overall or treatment-specific link between male aggressiveness and short-term delivery (aggression × treatment: χ^2^_6_ = 6.05, *P* = 0.42; main effect aggression: χ^2^_1_ = 0.72, *P* = 0.40), with no evidence for a difference between years (aggression × treatment × year: χ^2^_6_ = 9.54, *P* = 0.15).

**Figure 5:**
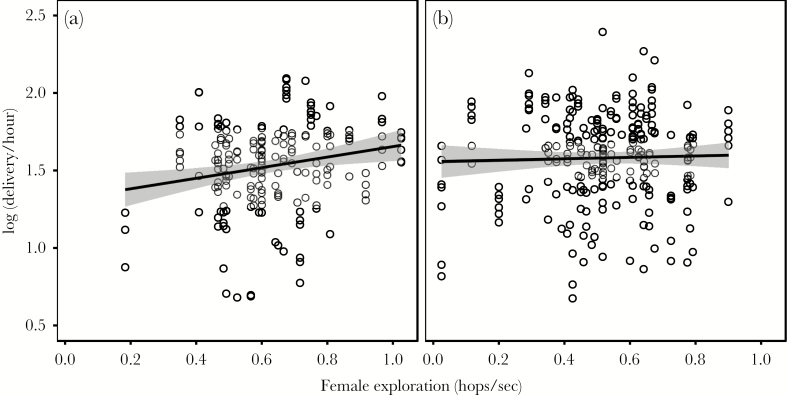
Relationship between female exploration behavior (number of movements per sec) and individual short-term delivery rates averaged per treatment in (a) 2010 and (b) 2011. C = control, B = blackbird, and WP = woodpecker presentation at the nestbox in the order presented in the experiment. Regression lines and 95% CI (shaded area) are fitted to raw data.

We found moderate support for a link between short-term delivery and exploration behavior that differed between years and sexes (exploration × year × sex × treatment: χ^2^_6_ = 12.64, *P* = 0.05). Rerunning models separately for both years and sexes revealed a nontreatment-specific effect for females in 2010 (main effect exploration: χ^2^_1_ = 4.60, *P* = 0.03; exploration × treatment: χ^2^_6_ = 5.31, *P* = 0.50). In this year, fast exploring females delivered food at higher rates compared with slow exploring ones (β = 0.40, 95% CI: 0.03, 0.71; Figure 5). For males in 2010, there was no support for a general (main effect exploration: χ^2^_1_ = 0.00, *P* = 0.90) and only weak evidence for a treatment-specific effect (exploration × treatment: χ^2^_6_ = 11.40, *P* = 0.09). In 2011, we did not find any support for a link between exploration and delivery in females (exploration × treatment: χ^2^_6_ = 8.46, *P* = 0.21; main effect exploration: χ^2^_1_ = 0.17, *P* = 0.68) or males (exploration × treatment: χ^2^_6_ = 5.31, *P* = 0.50; main effect exploration: χ^2^_1_ = 0.48, *P* = 0.49).

## DISCUSSION

Great tits adjusted provisioning behavior to current levels of nest predation threat in an apparently adaptive way. Parents delayed feeding when a model predator was presented near the nest box, which we interpret as a means of reducing the costs of a sudden increase in threat of nestling predation and adult injury. After the immediate threat ended, parents increased provisioning effort, which we interpret as a response to increased brood demand generated by the earlier interruption in provisioning. We found moderate evidence that these responses were linked with other nonparental risk-taking behaviors (i.e., aggressiveness and exploration behavior): the predicted relationships were only apparent in 1 year (and may thus depend on ecological conditions), and differed between the 2 behaviors and between the sexes.

### Immediate responses to increased perceived predation threat

As expected, great tit parents responded to the woodpecker by interrupting their provisioning behavior for long periods of time relative to either the black bird presentation or human disturbance (Figure 2a). This indicates that parents considered the woodpecker a potential threat even after initial effects of human disturbance and/or the sudden appearance of a bird close to their nest had decreased. Feeding latencies during blackbird presentation did not differ from treatments with short-term human disturbance only, implying that the feeding interruptions during blackbird presentations were attributable to the human disturbance. This shows that great tits were able to recognize features of the woodpecker specific to this type of nest predator: they were not merely responding to a simple disturbance. This finding is consistent with 3 previous bird studies investigating predator recognition during offspring provisioning (Curio 1975; Mutzel et al. 2013a; Moks et al. 2016).

Interestingly, parental feeding latencies during the blackbird treatment before and after the woodpecker presentation did not differ, even though nestlings were hungrier directly after the woodpecker treatment. This suggests that there were carry-over effects from the woodpecker presentation counteracting effects of increased nestling begging on parental feeding latency: the increased predation threat during the woodpecker treatment appeared to continue to cause delays in provisioning behavior even after the woodpecker was removed. This response might be adaptive, as rapidly resuming of provisioning following a disturbance might increase the likelihood of nest predation or adult injury, if the predator is likely to return. Alternatively, cumulative effects of repeated disturbance might have counteracted any effects of nestling begging. This latter explanation is unlikely, given that parents resumed provisioning faster during the last 2 controls, possibly in response to elevated begging of hungry nestlings following the feeding interruptions caused by the woodpecker and blackbird presentations.

The initial parental responses to disturbance in terms of latencies were similar for both years (Supplementary Figure S1a). This is surprising, given that nestlings in 2010 were in much poorer condition (Nicolaus et al. 2015; Mathot et al. 2017) and begged at considerably higher levels (Table 1). This implies that feeding interruptions should have had more detrimental effect on nestling growth and survival in 2010. The higher immediate nestling demand (“hunger”) in this year should thus have motivated parents to resume feeding sooner (Wright et al. 2010b). However, a brood in (long-term) poor condition (as in 2010) might actually be less valuable and parents might thus have been less willing to accept the costs of resuming provisioning in the presence of a potential nest predator (Montgomerie and Weatherhead 1988; Rytkönen 2002). Furthermore, parental feeding latencies might also vary between adults differing in risk-taking behavior under predation threat, rather than solely being a function of nestling hunger or brood value. Indeed, we found some evidence that feeding latencies were individually repeatable between years, suggesting that parental responses towards disturbance might be individual-specific.

### Variation in provisioning behavior in response to changes in perceived predation threat

Once parents returned to the nest in the presence of the woodpecker, they resumed normal provisioning behavior. This implies that they had habituated and that the level of perceived predation threat was relatively low (Rankin et al. 2009). However, this explanation does not include any effects of nestling begging. Indeed, nestlings begged at much higher levels during and directly after the predator presentation, which should have resulted in an increased provisioning rate (e.g., Ottosson et al. 1997; Kilner et al. 1999; Wright et al. 2010b). Taken together, these findings suggest that earlier exposure to a model woodpecker continued to affect parental provisioning behavior, but that this effect was counterbalanced by the effect of nestling begging. This demonstrates the importance of including nestling begging when investigating ecological effects on provisioning behavior.

Following predator model removal, parents increased provisioning, thus compensating for lost feeding opportunities. However, this compensation was mainly observed in the good year; evidence was weak in the poor year, perhaps because parents already provisioned closer to their maximum sustainable rates and thus were not able to further increase their level of provisioning (see Wright and Cuthill 1990). Moreover, prior to the experimental manipulation in 2010, offspring were already in relatively poor condition with a low survival probability (Mathot et al. 2017). In such a poor year, broods are of relatively low value (Montgomerie and Weatherhead 1988; Rytkönen 2002; Thünken et al. 2010), and parents might thus not be willing to increase effort and pay further costs of reproduction (e.g., Askenmo 1977; Gustafsson and Sutherland 1988; Dijkstra et al. 1990).

Parents increased provisioning after predator model removal, but did they manage to fully compensate for the lost feeding opportunities? The nestling begging data of the last 2 controls revealed that begging had returned to baseline levels by the end of the experiment. This indicates that parents had indeed compensated for the increased nestling need, at least with regard to short-term need. Interestingly, this was also true in the bad year, indicating that even the small increase in parental effort was enough to return nestling short-term nutritional needs to initial states. However, nestlings were still begging at much higher levels in 2010 (Supplementary Figure S1d), indicating that longer-term nestling need was still not adequately met in the poor year. This was confirmed by the finding that offspring survival probability after the experiment was much lower (2010: 76.3%; 2011: 98.2%) and that fledglings were also in worse condition (Nicolaus et al. 2015).

Parents appeared to adequately adjust provisioning to current levels of nestling short-term need, implying that increased provisioning effort after elevated levels of perceived predation threat was mediated through nestling begging intensity. An alternative explanation would be that parents increased their provisioning rates in order to lower the begging rate of nestlings and thus reduce the chance of attracting further predators to the nest. Yet, this could not explain the finding that parents maintained relatively high levels of provisioning even after nestling begging had returned to baseline levels during the last 2 controls (Figure 4). Although a short delay in parental responses might be an artifact of the statistical analysis (summarizing average behaviors during a 30-min treatment), this effect should have disappeared during the start of the last treatment when nestling begging was already back at baseline levels. There are 2 explanations for this finding. First, parents did not immediately respond to changes in begging intensity, but rather relied on a longer-term measure of nestling need (e.g., integrating begging intensity over a longer period). Few studies explicitly investigated the time span of nestling begging to which parents respond, but most studies suggest that parents respond relatively quickly to changes in nestling need, even on a visit-by-visit basis (e.g., Wright and Leonard 2002; McDonald et al. 2009a; Wright et al. 2010b; Kitamura et al. 2011). Alternatively, parents may not have used begging intensity (as we measured it) as the main cue for increasing provisioning effort. Indeed, several studies suggest that parents can or should ignore begging under certain ecological conditions (Davis et al. 1999; Thorogood et al. 2011; Caro et al. 2016) and begging playback experiments suggest they do (Santema et al. 2017). Parents might use other decision rules, for example, monitoring their long-term food delivery and compensating for temporary shortfalls regardless of begging. Alternatively, parents may use cues such as begging noise and acoustic structure, or the posture or movements of the offspring. Even if all these elements of begging covaried (Wright et al. 2002; Wright et al. 2010a), parents would have had access to the full information contained within all aspects of begging, which might not be sufficiently reflected in the variables we assessed.

### Individual-specific responses related to aggressiveness and exploration behavior

Investment in the current brood often comes at a cost of reducing parental survival (Lack 1947; Williams 1966; Clutton-Brock 1991; Royle et al. 2012). We thus predicted that individual differences in provisioning behavior should covary with variation in other risk-taking behaviors (Réale et al. 2010). Although we did not find a general link between aggressiveness, exploration behavior, and parental provisioning behavior, our study does suggest that such link might be present under certain conditions. In the more challenging year, fast-exploring females delivered more food. These individuals were perhaps investing more in current reproduction, as suggested in a previous study on blue tits where fast-exploring females, but not males, provisioned nestlings at higher rates (Mutzel et al. 2013b; Serrano-Davies et al. 2017). Our study further complements a previous finding in the same great tit population that fast- (compared to slow-) exploring females fledged more offspring in better condition when facing enlarged broods (Nicolaus et al. 2015). Altogether, this suggests that fast-exploring females might be more willing to pay the additional costs of increased provisioning in response to experimental brood size enlargement, whereas slow explorers might instead reduce their current reproductive success to protect future fitness interests (Cole and Quinn 2014).

But why did we find such a link only in one year? Individual differences in the willingness to forgo potential future breeding opportunities by investing more in the current brood may only become apparent under harsh environmental conditions (Montiglio et al. 2018; Nicolaus et al. 2019). Under such conditions, high levels of offspring provisioning can only be achieved at the expense of self-foraging or self-maintenance and/or by accepting a higher probability of mortality while foraging, thus resulting in a reduced future breeding potential. Such context-dependency might also explain why a previous study on another great tit population did not find any link between exploration and provisioning behavior (Patrick and Browning 2011).

Interestingly, the observed link was only found for female exploratory behavior; male aggressiveness or exploratory behavior did not affect nestling provisioning. Possibly, investment in current reproduction is reflected by different aspects of parental care for males versus females. For instance, males might invest more in nest and territory defense or seek extra-pair fertilizations instead of increasing parental care. More detailed behavioral observations are thus required to understand any sex-specific links between behaviors and life history (Hämäläinen et al. 2018).

This study did not assess long-term effects on parental survival and future reproductive success. Thus, we cannot exclude the possibility that individual variation in parental delivery rates, rather than reflecting individual differences in investment in the current brood, resulted from other differences among individuals, such as foraging ability. For instance, fast-exploring females might be better at finding and capturing food and might thus be able to provision at higher rates without investing more. However, this is contradicted by earlier studies suggesting that slow- and not fast-explorers are more flexible and better at locating new food sources, and thus better adapted to changeable and harsh environments (Verbeek et al. 1994; Drent and Marchetti 1999). It still remains to be shown that the detected differences in provisioning and exploration result from personality-related variation in how life-history trade-offs are resolved.

A previous study on the same great tit population found that fast-exploring females also produced larger clutches (Araya-Ajoy et al. 2016). However, it seems unlikely that the personality-related differences in female provisioning rates detected here are caused by differences in the initial investment in current reproduction. Although individual differences in parental “quality” can influence both clutch size and provisioning rate (see Wright and Cuthill 1992; Westneat et al. 2011), such patterns of covariance should have been removed by the brood size manipulation. Brood size manipulations are thus required to reveal the true scale of life-history trade-offs that would otherwise be obscured by individual differences in quality and/or resource acquisition (van Noordwijk and de Jong 1986; Reznick et al. 2000).

## CONCLUSIONS

By investigating parental provisioning decisions in response to prevailing levels of perceived predation threat, while at the same time closely monitoring changes in nestling need, we demonstrate that great tit parents adjust several aspects of provisioning behavior in an apparently adaptive way. During high levels of predation threat, parents interrupted nestling provisioning, thus reducing both the threat of predation to their nestlings and the threat of injury to themselves. Once the immediate threat had diminished, they compensated for lost feeding opportunities by increasing provisioning effort, thus meeting increased nestling demand caused by feeding disruptions. By the end of the experiment, nestling need had returned to baseline levels, indicating that parents had managed to fully compensate for increased short-term nestling needs (hunger). Surprisingly, parents continued to provision at elevated levels for an extended period of time even after they had apparently satisfied any increased nestling need. This challenges the assumption that parents adjust their provisioning effort solely towards changes in short-term nestling needs, and raises the question of what cues or decision rules parents use to adaptively adjust provisioning.

We further demonstrate that individual differences in exploration were linked to parental investment in the current brood, providing some evidence for the presence of individual differences in how individuals resolve life-history trade-offs (often referred to as pace-of-life or POLS theory; Réale et al. 2010; Dammhahn et al. 2018). These links were only apparent in one sex and in a year when parents seemed to work close to their limit. This suggests that experimental elevations in parental effort might be needed to reveal predicted individual differences that might otherwise be obscured by individual optimization of nest-site choice, brood size, etc. (also see Nicolaus et al. 2015). Moreover, we argue that an adequate test of the POLS-hypothesis requires investigating how supposedly costly behaviors and investment in current reproduction affect an individual’s survival and future reproductive output.

## FUNDING

This work was supported by the Max Planck Society (to B.K.) and CBD at the Department of Biology, Norwegian University of Science and Technology (NTNU), and partly supported by the Research Council of Norway through its Centers of Excellence funding scheme (project number 223257). A.M. was supported by the International Max Planck Research School for Organismal Biology (IMPRS), K.J.M. by postdoctoral fellowships from the Alexander von Humboldt Foundation (AvH) and the Natural Sciences and Engineering Research Council of Canada (NSERC), M.N. by an AvH Postdoctoral Fellowship, A.-L.O. by a PhD studentship from NTNU, and Y.G.A.-A. by IMPRS and a DAAD PhD scholarship. A.M., K.J.M., M.N., Y.G.A.-A., N.J.D., and B.K. were supported by the Max Planck Society.

## Supplementary Material

arz060_suppl_Supplement-Material

## Notes

Authors’ contributions: A.M. conceived the study. A.M., J.W., A.L.O., K.J.M., M.N., B.K., and N.J.D. designed the experiment. A.M., A.L.O., K.J.M., M.N., Y.G.A.-A., J.J.W., and N.J.D. performed the fieldwork. A.M. and A.L.O. analyzed the provisioning videos. A.M. performed the statistical analyses with input from N.J.D., J.W., K.J.M., Y.G.A.-A., and B.K. A.M. wrote the manuscript with input from N.J.D. and J.W., and all coauthors contributed to revisions.

## Notes

Data accessibility: Analyses reported in this article can be reproduced using the data provided by Mutzel et al. (2019).
