# Supplementary material for: Effects of manipulated levels of predation threat on parental provisioning and nestling begging
Source: Behav Ecol. 2019 Apr 24;30(4):1123–35. doi: 10.1093/beheco/arz060 (PMC6606999; doi:10.1093/beheco/arz060)
Supplement: arz060_suppl_Supplement-Material [file arz060_suppl_supplement-material.docx]

**Electronic Supplementary Material**

**Appendix S1. Effects of woodpecker and blackbird model identity on feeding latency and IVI**

To test for an effect of woodpecker model identity on feeding latency and IVIs, we ran likelihood ratio tests (LRTs) using the restricted maximum likelihood method (REML). The LRTs were constructed by comparing a linear mixed-effect model containing feeding latency or IVI as a fixed effect while also fitting random intercepts for nest identity and woodpecker model identity (‘model 1’) with a similar model where only random intercepts for nest identity were included (‘model 2’). For the IVIs models we additionally fitted random intercepts for individual identity. The LRTs did not reveal a significantly better fit of model 1 compared to model 2 for either feeding latency or IVIs, implying that woodpecker model identity did not explain a significant amount of the variation in feeding latency (AIC_model 1_ = 110.83, log *L*_model 1_ = -49.42; AIC_model 2_ = 108.83, log *L*_model 2_ = -49.42; χ^2^_1_ = 0, *P* = 1) and IVIs (AIC_model 1_ = 939.61, log *L*_model 1_ = -464.80; AIC_model 2_ = 937.71, log *L*_model 2_ = -464.80; χ^2^_1_ = 0.10, *P* = 0.75).

To test for an effect of blackbird model identity on feeding latency or IVIs, we run similar models with random intercepts for nest, individual and blackbird model identity. The LRTs did not reveal a significant better fit of model 1 compared to model 2 for both feeding latency and IVIs, implying that blackbird model identity did not explain a significant amount of the variation in feeding latency (AIC_model 1_= 252.07, log *L*_model 1_=-121.03; AIC_model 2_= 253.16, log *L*_model 2_= -122.58; χ^2^_1_ = 3.09, *P* = 0.08) and IVIs (AIC_model 1_ = 2631.3, log *L*_model 1_ = -1310.6; AIC_model 2_ = 2629.4, log *L*_model 2_ = -1310.7; χ^2^_1_ = 0.15, *P* = 0.69).

**Results from models where current brood size was replaced by natural brood size (number of hatchlings, mean-centered within year) and brood size manipulation (BSM; factor with 3 levels: reduced, control, enlarged).**

**Table S1. Sources of variation in latency, load size, short-term delivery and nestling begging with natural brood size and BSM.**

Estimates were derived from similar models as described for Table 1 (main text), but with current brood size replaced by natural brood size and brood size manipulation (BSM). Values presented are means (β) and 95% credible intervals (CIs). Effects that were strongly supported by the model (95% CIs not overlapping zero) are highlighted in bold face.

|  | Log(Latency) | Log(Load size) | Log(short-term delivery) | Begging |
| --- | --- | --- | --- | --- |
|  | β (95%CI) | β (95%CI) | β (95%CI) | β (95%CI) |
| Intercept | 2.54 (2.45, 2.64) | 0.28 (0.26, 0.30) | 1.48 (1.42, 1.54) | 4.79 (4.33, 5.21) |
| Treatment^a^ |  |  |  |  |
| C1 | **0.10** (0.03, 0.18) | 0.00 (-0.01, 0.02) | 0.00 (-0.05, 0.03) | 0.08 (-0.17, 0.31) |
| B1 | **0.15** (0.08, 0.24) | -0.01 (-0.02, 0.00) | -0.01 (-0.05, 0.03) | 0.14 (-0.11, 0.37) |
| WP | **0.31** (0.24, 0.40) | 0.00 (-0.02, 0.01) | -0.03 (-0.07, 0.02) | **0.39** (0.15, 0.66) |
| B2 | **0.13** (0.05, 0.20) | -0.00 (-0.02, 0.01) | 0.02 (0.00, 0.07) | **0.48** (0.23, 0.70) |
| C3 | **-0.11** (-0.19, -0.03) | -0.01 (-0.03, 0.00) | 0.02 (-0.01, 0.07) | 0.10 (-0.13, 0.33) |
| C4 | **-0.21** (-0.28, -0.12) | -0.01 (-0.02, 0.00) | 0.04 (0.00, 0.07) | -0.04 (-0.28, 0.20) |
| Sex male^b^ | -0.03 (-0.09, 0.02) | 0.01 (0.00, 0.02) | 0.00 (-0.03, 0.04) | **0.29** (0.21, 0.36) |
| Year 2011^c^ | -0.03 (-0.11, 0.05) | **0.09** (0.07, 0.11) | **0.09** (0.04, 0.14) | **-0.96** (-1.37, -0.52) |
| Natural brood size | **-0.06** (-0.09, -0.03) | 0.00 (-0.01, 0.00) | **0.03** (0.01, 0.05) | **0.17** (0.02, 0.31) |
| BSM^d^ |  |  |  |  |
| Reduced | 0.14 (-0.04, 0.23) | -0.01 (-0.04, 0.01) | -**0.09** (-0.14, -0.02) | -0.48 (-0.98, 0.08) |
| Enlarged | -0.05 (-0.16, 0.04) | -0.01 (-0.03, 0.01) | 0.01 (-0.06, 0.06) | **0.57** (0.06, 1.07) |
| *N* | 1208 | 8215 | 7119 | 7942 |

^a^ Reference category is treatment ‘C2’

^b^ Reference category is sex ‘female’

^c^ Reference category is year ‘2010’
^d^ Reference category is BSM ‘Control’

# Table S2. Sources of variation in IVI and long-term delivery with natural brood size and BSM in 2010 and 2011.

Estimates were derived from similar models as described for Table 2 (main text), but with current brood size replaced by natural brood size and brood size manipulation (BSM) for 2010 and 2011. Values presented are means (β) and 95% credible intervals (CIs). Effects that were strongly supported by the model (95% CIs not overlapping zero) are highlighted in bold face.

|  | Log(IVI) | Log(long-term delivery) |
| --- | --- | --- |
|  | β (95%CI) | β (95%CI) |
| 2010 |  |  |
| Intercept | 1.99 (1.90, 2.06) | 0.81 (0.70, 0.95) |
| Treatment^a^ |  |  |
| C1 | 0.01 (-0.06, 0.07) | -0.08 (-0.19, 0.01) |
| B1 | -0.01 (-0.08, 0.05) | **-0.14** (-0.23, -0.03) |
| WP | 0.00 (-0.06, 0.08) | **-0.24** (-0.34, -0.14) |
| B2 | -0.05 (-0.11, 0.01) | 0.01 (-0.08, 0.11) |
| C3 | -0.00 (-0.07, 0.06) | 0.08 (-0.04, 0.16) |
| C4 | -0.03 (-0.09, 0.04) | 0.08 (-0.02, 0.17) |
| Sex male^b^ | 0.03 (-0.03, 0.08) | 0.01 (-0.06, 0.10) |
| Natural brood size | -0.01 (-0.04, 0.01) | **0.06** (0.01, 0.11) |
| BSM^c^ |  |  |
| Reduced | 0.06 (-0.05, 0.14) | -0.07 (-0.27, 0.04) |
| Enlarged | 0.05 (-0.04, 0.13) | 0.00 (-0.16, 0.14) |
| *N* | 3351 | 556 |
| 2011 |  |  |
| Intercept | 2.12 (2.05, 2.20) | 0.98 (0.89, 1.07) |
| Treatment^a^ |  |  |
| C1 | 0.01 (-0.05, 0.08) | -0.05 (-0.13, 0.03) |
| B1 | -0.01 (-0.08, 0.05) | -0.08 (-0.15, 0.01) |
| WP | 0.02 (-0.05, 0.10) | **-0.33** (-0.39, -0.23) |
| B2 | -0.03 (-0.10, 0.03) | -0.06 (-0.14, 0.01) |
| C3 | **-0.11** (-0.16, -0.04) | **0.13** (0.05, 0.20) |
| C4 | **-0.10** (-0.15, -0.03) | **0.13** (0.04, 0.10) |
| Sex male^b^ | -0.02 (-0.07, 0.05) | 0.05 (-0.01, 0.11) |
| Natural brood size | **-0.04** (-0.06, -0.02) | **0.07** (0.04, 0.10) |
| BSM^c^ |  |  |
| Reduced | **0.08** (0.01, 0.18) | **-0.30** (-0.42, -0.20) |
| Enlarged | -0.06 (-0.14, 0.01) | 0.04 (-0.06, 0.16) |
| *N* | 3788 | 647 |

^a^ Reference category is treatment ‘C2’

# ^b^ Reference category is sex ‘female’ ^c^ Reference category is BSM ‘Control’

**Figure S1. Effect of experimental treatment on (a) feeding latency, (b) load size, (c) short-term delivery and (d) nestling begging in 2010 and 2011.**

C = control (C1 and C3: human disturbance at nestbox; C2 and C4: human disturbance at a distance of 20 m from nest), B = blackbird and WP = woodpecker presentations at the nestbox in the order presented in the experiment. Results are shown for the means and standard errors of the raw values.
